# Commentary: Cultural recycling of neural substrates during language evolution and development

**DOI:** 10.3389/fpsyg.2015.01507

**Published:** 2015-10-06

**Authors:** Patrick C. Trettenbrein

**Affiliations:** ^1^Language Development & Cognitive Science Unit, Department of English Studies, University of GrazGraz, Austria; ^2^Department of Linguistics, University of GrazGraz, Austria

**Keywords:** evolution of language, language development, Universal Grammar (UG), neural recycling, cultural recycling, linguistics, cognitive science, cognitive neuroscience

In their contribution to *The Cognitive Neurosciences V*, the most recent addition to the popular series of works of reference among cognitive (neuro-)scientists, Christiansen and Müller (2015) discuss the role of phylogenetically ancient neural circuits in the evolution of the human language faculty (FL) and its ontogenetic development from the perspective of what they refer to as “cultural recycling.” While I am in principle highly sympathetic to any endeavor that addresses “Darwin's problem” (Boeckx, 2009) within the conceptual framework of “neural recycling” (e.g., Dehaene and Cohen, 2007), it occurs to me that Christiansen and Müller's outright dismissal of Universal Grammar (UG) is premature and ill-founded, as they reiterate misconceptions regarding evolutionary biology and UG that are common in the literature (e.g., Dunbar, 2003; Christiansen and Chater, 2008; Christiansen et al., 2009; Evans and Levinson, 2009; Chater and Christiansen, 2010).

Christiansen and Müller (2015) rightly call attention to the fact that the evolution of UG, that is seemingly (highly) arbitrary linguistic principles, by means of natural selection as, for example, put forward by Pinker and Bloom (1990) actually delineates a next to impossible scenario for human cognitive phylogeny. Clearly, the arbitrariness of UG principles would constitute a “moving target” for natural selection, that is a target that could hardly, if at all, have been selected for (Chater et al., 2009). The authors present this conclusion as evidence against the existence of UG and base their case for “cultural evolution” on it. Yet, in so doing they fail to fully explore the consequences of this claim. As Berwick (2009) already pointed out, an independent movement in theoretical linguistics has come to strikingly similar conclusions and has carried on where Christiansen and Müller (2015) seemingly chose to leave off.

The assertion that FL did not “evolve” (in a neo-Darwinian sense) might appear somewhat bizarre at first, but the idea that natural selection is not the only force in evolution was already acknowledged by Darwin himself (in later editions of *Origin of Species*) and has been gaining widespread acceptance among biologists in the last two or so decades (Fodor and Piattelli-Palmarini, 2010/2011). Along these lines the so-called language as a “spandrel” (Gould and Lewontin, 1979) scenario has repeatedly and plausibly been put forward in the literature (e.g., Hauser et al., 2002; Fitch et al., 2005; Bolhuis et al., 2014). In essence, the argument is that FL, being universal among humans (Anderson and Lightfoot, 2000; Berwick et al., 2013; Hauser et al., 2014), arose “recently” and “suddenly” (Tattersall, 2009, 2013), a stance that deems large parts of FL to be exaptations (Fitch, 2010, 2011).

Such an exaptationist train of thought seems to me to be, in principle, highly compatible with Christiansen and Müller's goal of painting a picture of FL within the conceptual framework of neural recycling during phylogeny. In the terminology of Hauser et al. (2002), the faculty of language in a broad sense (FLB) should be expected to have inherited properties of the evolutionary ancient systems of which it is composed so that the novelty of the narrow language faculty (FLN) might even come down to nothing else than the way in which it links these ancient systems together. Thus, the object of interest is the evolution of language as “[…] a computational cognitive mechanism that has hierarchical syntactic structure at its core […]” (Bolhuis et al., 2014, p. 1). Pressingly, we find that the design of this mechanism is often ill-suited for communicative purposes, instead adhering to principles of efficient computation (e.g., Chomsky, 2011).

Because UG cannot have evolved through natural selection Christiansen and Müller introduce “cultural recycling” of preexisting neural structures as a supposed alternative. Despite the authors' statement that “Without our brains, there would be no language.” (Christiansen and Müller, 2015, p. 676), they nevertheless assert that as the brain did not adapt for language, language(s) instead must have adapted to human brains through “cultural evolution.” Given that there is no way for an I-language to “exist” apart from the human mind/brain (Chomsky, 1986), it is completely unclear how a language that did not “fit” the human brain could ever have existed (the hypothetical realm of the theorist's design space notwithstanding). Consequently, the “close fit” between language design and the human brain is, contrary to Christiansen and Müller's assessment, neither surprising nor in need of explanation.

As a case in point, given the pace of language change (i.e., “cultural evolution”), all properties of language that counteract communicative efficiency (Christiansen and Müller's “socio-pragmatic considerations”) should long have been eliminated if there was no UG, provided that FL emerged roughly 100,000 years ago (Tattersall, 2009, 2013; Bolhuis et al., 2014). Yet contrary to Christiansen and Müller's conjecture this is not the case. The corresponding conclusion is that natural languages do not *evolve* in a (neo-)Darwinian sense, instead they *change* (Chomsky, 2011) within a rather narrow framework of possible variation provided by UG (currently best captured by the concept of parametric variation; see Baker, 2001). This fixed framework concerns linguistic features as opposed to their culturally determined values (Berwick, 2009), a distinction that the authors fail to make. Thus, accepting that the linguistic genotype is fixed (Anderson and Lightfoot, 2000; Berwick et al., 2013; Hauser et al., 2014), as it would seem that Christiansen and Müller (2015) do, language change becomes irrelevant for the study of the phylogeny of FL, and the role of “cultural evolution” is rendered even more obscure.

Christiansen and Müller's (2015) dismissal of UG in part also rests upon a discussion of the multiple cognitive functions that have credibly been localized to Broca's area (i.e., Brodmann area 44/45), a canonical “language region.” But from an exaptationist point of view this is not surprising, after all, the evolutionary change that created FL is conceived as minimal so that we should expect recycling of the same neural tissue for different functions. Nevertheless, functional specificity for language has been demonstrated (Musso et al., 2003; Fedorenko et al., 2011), and the processing of hierarchical structure as a core linguistic operation has successfully been isolated from other functions attributed to Broca's area, such as working memory (Makuuchi et al., 2009). Furthermore, observed variability in the neural basis of the world's languages is generally highly restricted, whereas linguistic function (e.g., syntactic, lexico-semantic, etc.) wins out over form (i.e., encoding) in all known respects (Friederici and Rüschenmeyer, 2006; Friederici, 2011), even independent of modality (Emmorey, 2006).

Unfortunately, available neuroimaging methods are not (yet) capable of capturing minute details with respect to (online) spatial and temporal resolution (Friederici and Rüschenmeyer, 2006; Kemmerer, 2015). However, everything currently known about the encoding of information in single neurons and cell assemblies (Quiroga et al., 2005; Sterling and Laughlin, 2015), as well as the fundamental principles on which much simpler nervous systems realize cognitive functions (Gallistel, 2009), points toward arbitrariness. While coding and computation are currently best understood in input-systems assigning “some order to our experience” (Anderson and Lightfoot, 2000, p. 15), such as the visual system famously studied by Hubel and Wiesel (1962), there is no reason to suppose that higher cognitive functions should be an exception. In general, the “atoms” of neural computation (Marcus et al., 2014) remain largely unknown and the granularity mismatch problem (Poeppel and Embick, 2005/2013) pertains, but efforts to ground linguists' abstractions in neurophysiology are under way (Friederici and Singer, 2015).

What then to say in conclusion? The idea that UG might have arisen through the recombination of already existing neural systems with (highly) arbitrary features from which new (language-specific) ones could have emerged remains credible in the light of contemporary neuroscience. The basic premise of Christiansen and Müller's (2015) proposed solution for Darwin's problem is interesting and fully compatible with recent independent efforts of theoretical linguists, but as yet I do not see any reason to throw out the baby with the bathwater by either dismissing UG or intermingling the study of language change with the study of the evolution of FL.

## Conflict of interest statement

The author declares that the research was conducted in the absence of any commercial or financial relationships that could be construed as a potential conflict of interest.
